# Temperament and Impulsivity Predictors of Smoking Cessation Outcomes

**DOI:** 10.1371/journal.pone.0112440

**Published:** 2014-12-04

**Authors:** Francisca López-Torrecillas, José C. Perales, Ana Nieto-Ruiz, Antonio Verdejo-García

**Affiliations:** 1 Department of Personality, Assessment and Psychological Treatment, University of Granada, Granada, Andalucía, Spain; 2 Brain, Mind and Behavior Research Center, University of Granada, Granada, Andalucía, Spain; 3 Department of Experimental Psychology, University of Granada, Granada, Andalucía, Spain; 4 Red de Trastornos Adictivos, University of Granada, Granada, Andalucía, Spain; 5 Occupational Medicine Area (Prevention Service), University of Granada, Granada, Andalucía, Spain; 6 Institute of Neuroscience F. Oloriz, Universidad de Granada, Granada, Andalucía, Spain; 7 School of Psychological Sciences, Monash University, Melbourne, Victoria, Australia; Brown University, United States of America

## Abstract

**Aims:**

Temperament and impulsivity are powerful predictors of addiction treatment outcomes. However, a comprehensive assessment of these features has not been examined in relation to smoking cessation outcomes.

**Methods:**

Naturalistic prospective study. Treatment-seeking smokers (n = 140) were recruited as they engaged in an occupational health clinic providing smoking cessation treatment between 2009 and 2013. Participants were assessed at baseline with measures of temperament (Temperament and Character Inventory), trait impulsivity (Barratt Impulsivity Scale), and cognitive impulsivity (Go/No Go, Delay Discounting and Iowa Gambling Task). The outcome measure was treatment status, coded as “dropout” versus “relapse” versus “abstinence” at 3, 6, and 12 months endpoints. Participants were telephonically contacted and reminded of follow-up face to face assessments at each endpoint. The participants that failed to answer the phone calls or self-reported discontinuation of treatment and failed to attend the upcoming follow-up session were coded as dropouts. The participants that self-reported continuing treatment, and successfully attended the upcoming follow-up session were coded as either “relapse” or “abstinence”, based on the results of smoking behavior self-reports cross-validated with co-oximetry hemoglobin levels. Multinomial regression models were conducted to test whether temperament and impulsivity measures predicted dropout and relapse relative to abstinence outcomes.

**Results:**

Higher scores on temperament dimensions of novelty seeking and reward dependence predicted poorer retention across endpoints, whereas only higher scores on persistence predicted greater relapse. Higher scores on the trait dimension of non-planning impulsivity but not performance on cognitive impulsivity predicted poorer retention. Higher non-planning impulsivity and poorer performance in the Iowa Gambling Task predicted greater relapse at 3 and 6 months and 6 months respectively.

**Conclusion:**

Temperament measures, and specifically novelty seeking and reward dependence, predict smoking cessation treatment retention, whereas persistence, non-planning impulsivity and poor decision-making predict smoking relapse.

## Introduction

An outstanding challenge for smoking cessation interventions is the detection of individual differences associated with poor treatment outcomes, especially in relation with long-term relapse [1], [2]. Individual differences in temperament and impulsivity are increasingly recognised as powerful predictors of addiction treatment outcomes, including smoking cessation outcomes [3]. Temperament refers to stable dispositions determining motivated behaviour [4]. Cloninger's model posits the existence of four major dimensions of temperament: novelty seeking, harm avoidance, reward dependence and persistence [4]. There is evidence that novelty seeking and harm avoidance are increased and persistence is decreased in heavy smokers [5]. However, only novelty seeking has been studied in relation to smoking cessation outcomes. Specifically, higher levels of novelty seeking associate with lower abstinence rates at mid-term (2 to 6 months) and long-term (12 months) follow-ups [6], [7]. This association is at least partly explained by poorer treatment retention in high novelty seekers [7]. Impulsivity refers to the tendency to engage in rapid behaviour without adequate forethought about the potential consequences of this behaviour [8]. Current theories differentiate between trait and cognitive aspects of impulsive behaviour, with trait aspects representing personality features leading to impulsive outcomes, and cognitive aspects representing the moment-to-moment function of the cognitive processes that regulate impulse control [9], [10]. Trait aspects include motor, attentional and non-planning impulsivity [11] and cognitive aspects include response inhibition, delay discounting and reward/punishment-based decision-making skills [11]. Several studies have shown that separate aspects of trait and cognitive impulsivity are significantly associated with smoking cessation outcomes. Specifically, higher levels of trait impulsivity associate with short-term and mid-term smoking relapse (i.e., 1 week and 3 months respectively) [12], [13]. Further, poorer performance on response inhibition or delay discounting measures associates with mid-term smoking relapse (3 to 6 months) [13], [14]. However, no studies to date have thoroughly mapped the link between the multimodal (i.e., trait and cognitive) and multifaceted (i.e., separate dimensions of trait and cognitive) aspects of impulsivity and smoking cessation outcomes.

In addition, the broader literature on the link between individual differences and addiction treatment outcomes has provided growing insights about the association between temperament, impulsivity and treatment outcomes, which have not yet been examined in smoking cessation studies. Firstly, there is evidence to suggest that the link between specific aspects of temperament and impulsivity and addiction treatment outcomes differ as a function of the type of outcomes (i.e., retention versus relapse) and the timing of endpoints. For example, in opiate using populations higher levels of novelty seeking are associated with better treatment commitment during the first weeks of combined pharmacological and behavioural interventions, but lower treatment retention by 3-month follow-up [15]. It is plausible to think that other temperament dimensions (i.e., persistence) may contribute to explain why novelty seekers disengage from addiction treatment outcomes in the long-term [16]. Further, in stimulant using populations, higher levels of impulsivity are associated with better retention in combined pharmacological and contingency management interventions [17], but lower abstinence rates during and following interventions [18]. Both findings are relevant for smoking treatment, since combined pharmacological and behavioural interventions for smoking are typically associated with good short-term retention rates yet high levels of long-term relapse [1]. Furthermore, in multimodal assessments of cognitive impulsivity the dimension of decision-making seems to be a significantly stronger predictor of alcohol and opiates relapse relative to other impulsivity indices [19], [20]. This association is particularly robust in outpatient settings [21] and seems to generalise to long-term outcomes [22]. These findings are as well relevant for smoking cessation treatment, since interventions of choice are outpatient-based, and mainly plagued by long-term relapse [1], [2]. Therefore, temperament and impulsivity are linked to smoking cessation outcomes, but there is no comprehensive mapping of which dimensions of these features are relevant (versus irrelevant) to predict different outcomes (retention and relapse) at different time points (short-term versus mid-term versus long-term endpoints). Moreover, trait and cognitive measures of impulsivity are often poorly correlated, and there is growing interest in understanding their relative contribution to outcomes in multiple regression approaches [23]. In this study, we conducted multiple measures of temperament and impulsivity at the onset of smoking cessation treatment including pharmacological and behavioural components, and utilised these measures to predict retention and relapse at 3, 6 and 12 months endpoints.

Temperament and impulsivity are theoretically different constructs, with the former emphasising motivational tendencies and the latter emphasising the degree of control over these motivational tendencies [24]. Moreover, both constructs may have different implications for addiction treatment since temperament is more stable across time [25] whereas impulsivity -even trait impulsivity- is amenable to addiction interventions [26]. Therefore, we conducted separate prediction models for temperament measures (novelty seeking, harm avoidance, reward dependence and persistence) and trait and cognitive impulsivity measures (motor, attention and non-planning, traits and cognitive, delay discounting and decision-making skills). In agreement with existing studies, we hypothesised that higher novelty seeking and persistence would be associated with greater short-term and long-term retention and relapse respectively and that poor performance on the cognitive impulsivity component of decision-making would be associated both with greater short-term and long-term relapse.

## Methods

### Design

We conducted a naturalistic prospective study during the course of a smoking cessation treatment intervention. We utilised baseline psychometric assessments to predict treatment status at three endpoints: 3 months after treatment commencement; 6 months after treatment commencement; and 12 months after treatment commencement. Treatment-seeking smokers were recruited as they engaged in an occupational health service that provides smoking cessation treatment including pharmacological and behavioural components between September 2009 and September 2013. The treatment consisted on three consecutive phases: (1) psychoeducation and counselling to reduce smoking; (2) prescription of the drug varenicline, in alignment with the Food and Drug Administration's guidelines [27], [28] and (3) training of relapse prevention strategies. The participants' compliance with treatment was clinically monitored through 3 follow-up face to face sessions conducted at 3, 6 and 12 months after treatment commencement. The researchers telephonically contacted the participants before each of these follow-up sessions in order to enquire about their current treatment status: had they discontinued treatment (dropout), had they relapsed but intended to continue treatment (relapse), or were they abstinent and intended to continue treatment (abstinence). The treatment program permitted that participants originally classified as dropouts resumed treatment at a later stage (i.e., they re-entered phase (3), and clinicians focused on reconsolidating relapse prevention strategies). We adapted the research design to the treatment program, and therefore participants classified as dropouts at the 3-month endpoint could be re-classified as relapse or abstinence at the 6 or 12 months endpoints. Therefore, the three endpoints are discrete-time events.

### Participants

One hundred and forty smokers were recruited across 3 years. The demographic and smoking behaviour characteristics of the sample are displayed in Table 1. Participants were eligible if they were current smokers, aged above 18 years old, and were employed by the service provider (Universidad de Granada). The exclusion criteria were as follows: history of major mental disorders (i.e., major depression, psychosis) or current psychotropic medication for psychiatric symptoms, concurrent dependence on other substances (cocaine, heroin, alcohol, etc.), and current use of prescription medications that are incompatible with the pharmacological treatment used in the therapy.

**Table 1:** Baseline demographic and smoking characteristics of the participants.

Variables	Scores
Age (mean and SD)	47.36 (8.19)
Gender (N)	
Male (Female)	55 (85)
Education (N)	
Primary/Secondary	76
Tertiary/Ph.D.	64
Career (N)	
Administrative and Service Personnel	113
Academics/Researchers	27
Years of smoking addiction (mean and SD)	28.49 (10.09)
Number of daily cigarettes (mean and SD)	19.85 (9.17)
Fagerström test scores (mean and SD)	4.65 (2.32)
Cigarettes Brand (N)	
Blonde	120
Black Tobacco	12
Rolling	8

### Setting

The Occupational Health Prevention Service of the Universidad de Granada (Spain). The service includes a smoking clinic, managed by two physicians and one psychologist, which provides specialised treatment for smoking cessation including pharmacological (i.e., varenicline) and behavioral change (counselling+relapse prevention) components.

### Procedures

The study was approved by the Human Ethics Committee of the Universidad de Granada. All clients commencing treatment at the Occupational Medicine Prevention Service (n = 164) were invited to participate in a prospective study assessing personality and cognition in relation to smoking behaviour during the first contact with the clinic. The clients that provided informed consent by signed and met inclusion and exclusion criteria were scheduled for a baseline assessment before treatment onset. Subsequent telephone contact points were scheduled at 3, 6, and 12 months after this baseline assessment, before each of the treatment's follow-up sessions. Participants were telephonically contacted by an independent assessor (blind to study purpose and methods) at each endpoint (3, 6 and 12 months) in order to monitor their compliance with the treatment and their willingness to participate in the follow-up face to face assessments. The participants that failed to answer the phone calls or self-reported discontinuation of treatment and failed to attend the upcoming follow-up session were coded as dropouts. The participants that self-reported continuing treatment, and successfully attended the upcoming follow-up session were coded as either “relapse” or “abstinence”, based on the results of smoking behavior self-reports cross-validated with co-oximetry hemoglobin levels. This definition of outcomes (dropout versus relapse versus abstinence) has been deemed optimal for research designs embedded in smoking cessation treatment programs [29], [30]. Outcome data (dropout versus relapse versus abstinence) was obtained for 140 participants at 3-months, 123 participants at 6 months, and 112 participants at 12 months. The primary outcome variable (i.e., the classification of participants as dropout or relapse or abstinence) was assessed and coded independently at each of the endpoints.

### Measures

#### Semi-structured interview for smokers [31]

This survey provides information about socio-demographic data, family history, smoking duration, brand of cigarettes, and level of dependence.

#### Fargerström Test for Nicotine Dependence [32]

This test is composed of 6 items with two or four response alternatives. Its factorial structure is consistent [33] and there is a Spanish version of the test [34].

#### Temperament and Character Inventory Revised (TCI-R) [4]

This self-report questionnaire consists of 240 items which participants have to rate in a 5-point Likert scale. The items are grouped in four main temperament dimensions (Novelty Seeking, Harm Avoidance, Reward Dependence, and Persistence) and three character dimensions (Self-directedness, Cooperativeness, and Self-transcendence).

#### Barratt Impulsiveness Scale (BIS-11) [11]

This self-report scale consists of 30 items reflecting a collection of typical impulsivity manifestations. Participants have to rate to what extent these manifestations apply to them in terms of frequency: never or rarely, occasionally, often, and always or almost always (scoring from 0 to 4). The main dependent variable was the total impulsivity score, and three subscale scores: cognitive, motor, and non-planning impulsiveness.

#### Go/No Go [35]

The task consisted of 60 trials. In the first 30 trials (pre-switch), participants were asked to press a key as quickly as they could whenever the go stimulus (a letter) was presented, and to withhold the response when the no-go stimulus (a different letter) was presented. In the second 30 trials of the task (post-switch), participants were asked to respond to the previously no-go stimulus and not to respond to the previously go stimulus. The proportion of go vs. no-go trials on both phases (pre- and post-switch) was 7/3. The inter-stimulus interval (ISI) was set at 100 ms, and each stimulus was presented during 1000 ms. Auditory feedback (one of two distinctive sounds) was provided after each response to indicate whether that response had been right or wrong. Responses were coded as hits (responding in presence the go trial), false alarms (responding in presence of the no-go trial), misses (not responding in presence of the go trial), and correct rejections (not responding in presence of the no-go trial). The main dependent variable from this test was the false alarm rate, computed as the ratio between the number of false alarms and the total number of no-go trials (#false alarms+#correct rejections).

#### Delay-discounting questionnaire (DDT) [36]

This is a monetary-choice questionnaire asking for individual preferences between smaller, immediate rewards and larger, delayed rewards varying on their value and time to be delivered. The questionnaire is composed of a fixed set of 27 choices; the amounts of money and delays used in all 27 trials are reported in [36]. We calculated the area under the curve (AUC) [37] as the main dependent variable from this measure. The AUC was calculated for the range of reward magnitudes included in the questionnaire (small –Euro 25 to 35; medium –Euro 50 to 60; and large –Euro 75 to 85), according to the formula (x2−x1) [(y1−y2)/2], where x1 and x2 are successive delays, and y 1 and y 2 are the subjective values associated with these delays.

#### Iowa Gambling Task, original version [38]

This is a computer task measuring reward/punishment based decision-making. It involves four decks of cards (A, B, C and D). Each time a participant selects a card, a specified amount of play money is awarded. However, interspersed among these rewards, there are probabilistic punishments (monetary losses). Two of the decks of cards (A and B) produce high immediate gains; however, in the long run, they will take more money than they give, and are thus considered disadvantageous. The other two decks (C and D) are considered advantageous, as they result in small, immediate gains, but will yield more money than they take in the long run. The performance measure was the net score calculated by subtracting the number of disadvantageous choices (decks A and B) from the number of advantageous choices (decks C and D).

### Main outcome measure

Treatment compliance, defined as “treatment dropout” (i.e., non-response to the phone call or negative response to the query of whether they continue in treatment and non-attendance to the follow-up session) versus “smoking abstinence” versus “smoking relapse”, as measured by cross-validated self-reports of the last 3 months and co-oximetry hemoglobin levels sensitive to the last 24 hours. A positive self-report of smoking in the last 3 months and/or a CO level superior to 10 ppm was utilised to define relapse versus abstinence.

### Statistical analyses

We performed two series of multinomial regression analyses including two sets of predictors: (1) temperament scores (TCI's scores on dimensions of novelty seeking, harm avoidance, reward dependence and persistence), and (2) impulsivity scores from trait measures (BIS motor, cognitive and non-planning impulsivity) and cognitive tests (Go/No Go false alarms, Delay Discounting area under the curve and Iowa Gambling Task net scores). The dependent variable was the type of outcome, representing whether participants had (1) dropped out from treatment (Treatment Dropout), (2) relapsed during treatment (Relapse), or (3) maintained abstinence during treatment (Abstinence). This outcome measure was coded at each of the follow-up time points: 3 months, 6 months, and 12 months. In all the regression models, we set “maintained abstinence during treatment” (Abstinence) as the reference category, such that models tested which variables were significantly associated with Treatment Dropout or Relapse, relative to Abstinence. Multinomial regression analyses are the best-suited approach for the study design as they model the impact of several predictors on outcomes of multiple categories [39]. In addition, we conducted sensitivity analyses utilising bivariate logistic regression models separate for dropouts versus completers, and for abstinence versus relapse.

## Results

### Number of cases that drop-out, relapse or maintain abstinence at each of the follow-up time points

At the 3-month follow-up (*n* = 140), 32 participants had dropped out from treatment, 27 participants had relapsed during treatment, and 81 participants had maintained abstinence during treatment. At the 6-month follow-up (*n* = 123), 30 participants had dropped out from treatment, 37 participants had relapsed during treatment, and 56 participants had maintained abstinence during treatment. At the 12-month follow-up (*n* = 112), 28 participants had dropped out from treatment, 40 participants had relapsed during treatment, and 44 participants had maintained abstinence during treatment. Participants classified in each of these categories (dropout versus relapse versus abstinence) at each of the three follow-ups did not significantly differ in demographic or baseline smoking behaviour characteristics (see Table 2).

**Table 2:** Baseline demographic and variables related to cigarette smoking of the participants classified in each of these categories (dropout versus relapse versus abstinence).

Variables	DROPOUT	RELAPSE	ABSTINENT	*F/χ^2^*	*p*
***3-month follow-up***					
**Age (mean and SD)**	47.53 (8.56)	47.74 (8.48)	47.17 (8.05)	.056	.945
**Gender (N)**					
*Male (Female)*	11 (21)	11 (16)	33 (48)	2	.811
**Education (N)**					
*Primary/Secondary*	18	16	42	.512	.774
*Tertiary/Ph.D.*	14	11	39		
**Career (N)**				1.071	.585
*Administrative and Service Personnel*	27	20	66		
*Academics/Researchers*	5	7	15		
**Years of smoking addiction (mean and SD)**	29.94 (10.65)	29.44 (10.21)	27.60 (9.86)	.759	.470
**Number of cigarettes per day (mean and SD)**	22.00 (10.43)	20.85 (8.09)	18.67 (8.89)	1.735	.180
**Fagerström scores (mean and SD)**	4.75 (2.31)	4.63 (2.39)	4.62 (2.32)	.038	.962
**Cigarettes Brand (N)**					
*Blonde*	28	23	69	.657	.957
*Black Tobacco*	2	3	7		
*Rolling*	2	1	5		
***6-month follow-up***					
**Age (mean and SD)**	47.13 (8.62)	47.43 (9.68)	47.04 (6.91)	.026	.974
**Gender (N)**					
*Male (Female)*	11 (19)	15 (22)	25 (31)	.531	.767
**Education (N)**				.412	.814
*Primary/Secondary*	16	21	28		
*Tertiary/Ph.D.*	14	16	28		
**Career (N)**				.328	.849
*Administrative and Service Personnel*	25	29	44		
*Academics/Researchers*	5	8	12		
**Years of smoking addiction (mean and SD)**	29.87(10.98)	28.35 (11.75)	27.50(8.81)	.516	.598
**Number of cigarettes per day (mean and SD)**	21.83(10.47)	19.78 (7.90)	18.55 (8.74)	1.312	.273
**Fagerström scores (mean and SD)**	4.80 (2.37)	4.68 (2.16)	4.38 (2.30)	.398	.672
**Cigarettes Brand (N)**					
*Blonde*	26	32	47	1.828	.767
*Black Tobacco*	2	4	4		
*Rolling*	2	1	5		
***12-month follow-up***					
**Age of the respondents (mean and SD)**	46.62 (8.84)	48.55 (8.44)	45.77 (7.44)	1.224	.298
**Gender (N)**				.674	.714
*Male (Female)*	10 (18)	17 (23)	20 (24)		
**Education (N)**				1.826	.401
*Primary/Secondary*	15	23	19		
*Tertiary/Ph.D.*	13	17	25		
**Career (N)**				1.749	.417
*Administrative and Service Personnel*	24	29	35		
*Academics/Researchers*	4	11	9		
**Years of smoking addiction (mean and SD)**	29.39 (11.22	29.50 (10.62)	26.41 (9.50))	1.158	.318
**Number of cigarettes per day (mean and SD)**	21.61 (10.73)	19.98 (7.82)	19.16 (9.10)	.620	.540
**Fagerström scores (mean and SD)**	4.57 (2.27)	4.75 (2.30)	4.48 (2.26)	.153	.858
**Cigarettes Brand (N)**					
*Blonde*	24	35	37	6.092	.192
*Black Tobacco*	2	5	2		
*Rolling*	2	0	5		

### Multinomial regression models of temperament measures predicting follow-up outcome (Drop-out and Relapse vs. Abstinence)

For the 3-month follow-up outcome, the model including TCI scores showed satisfactory fit, *χ^2^* = 25.15, *d.f.* = 8, *p*<0.01, explaining 19% of variance, *Nagelkerke pseudo-R^2^* = 0.19. Inspection of parameter estimates showed that the dimension of novelty seeking, *Wald statistic* = 9.40, *p* = 0.002, and the dimension of reward dependence, *Wald statistic* = 5.28, *p* = 0.02, were significantly and directly associated with Treatment Dropout. None of the temperament dimensions were significantly associated with relapse at 3 months.

For the 6-month follow-up outcome, the model including TCI scores showed satisfactory fit, *χ^2^* = 31.79, *d.f.* = 8, *p*<0.001, explaining 26% of variance, *Nagelkerke pseudo-R^2^* = 0.26. Inspection of parameter estimates showed that the dimensions of novelty seeking, *Wald statistic* = 8.75, *p* = 0.003, reward dependence, *Wald statistic* = 3.93, *p* = 0.047, and persistence, *Wald statistic* = 6.45, *p* = 0.01, were significantly and directly associated with Treatment Dropout. Moreover, the dimension of persistence was also significantly associated with Relapse at 6 months, *Wald statistic* = 5.90, *p* = 0.02.

For the 12-month follow-up outcome, the model including TCI scores showed satisfactory fit, *χ^2^* = 24.55, *d.f.* = 8, *p*<0.01, explaining 22% of variance, *Nagelkerke pseudo-R^2^* = 0.22. Inspection of parameter estimates showed that the dimensions of novelty seeking, *Wald statistic* = 6.67, *p* = 0.01, and reward dependence, *Wald statistic* = 3.83, *p* = 0.05, were significantly and directly associated with Treatment Dropout. None of the temperament dimensions were significantly associated with Relapse at 12 months (see Table 3).

**Table 3:** Multinomial regression models testing the association between TCI temperament dimensions and smoking cessation treatment dropout and relapse at the 3-month, 6-month and 12-month endpoints.

Temperament Predictors	Three months						Six months						Twelve months					
	Dropout			Relapse			Dropout			Relapse			Dropout			Relapse		
	Wald	p	95% CI	Wald	p	95% CI	Wald	p	95% CI	Wald	p	95% CI	Wald	p	95% CI	Wald	p	95% CI
Novelty seeking	9.40	.002*	1.02–1.10	0.40	.527	.98–1.05	8.75	.003*	1.02–1.11	0.85	.357	.98–1.05	6.67	.010*	1.01–1.11	0.01	.903	.97–1.04
Harm avoidance	0.31	.580	.98–1.03	0.70	.402	.99–1.04	0.46	.497	.98–1.04	1.18	.277	.99–1.04	0.87	.352	.98–1.05	0.88	.349	.99–1.04
Reward dependence	5.28	.022*	1.01–1.07	0.31	.578	.98–1.04	3.93	.047*	1.00–1.08	0.06	.797	.96–1.03	3.83	.050	1.00–1.08	0.04	.845	.97–1.03
Persistence	2.35	.125	.99–1.05	1.57	.210	.99–1.05	6.45	.011*	1.01–1.08	5.90	.015*	1.01–1.07	2.74	.098	1.0–1.07	1.75	.186	.99–1.05

*p<0.05.

### Multinomial regression models of impulsive measures predicting follow-up outcome (Drop-out and Relapse vs. Abstinence)

For the 3-month follow-up outcome, the model including trait and cognitive impulsivity scores showed satisfactory fit, *χ^2^* = 60.31, *d.f.* = 12, *p*<0.001, explaining 41% of variance, *Nagelkerke pseudo-R^2^* = 0.41. Inspection of parameter estimates showed that the BIS dimension of non-planning impulsivity was significantly and positively associated with both Treatment Dropout, *Wald statistic* = 6.02, *p* = 0.01, and Relapse, *Wald statistic* = 5.34, p = 0.02.

For the 6-month follow-up outcome, the model including trait and cognitive impulsivity scores showed satisfactory fit, *χ^2^* = 64.39, *d.f.* = 12, *p*<0.001, explaining 46% of variance, *Nagelkerke pseudo-R^2^* = 0.46. Inspection of parameter estimates showed that the BIS dimension of non-planning impulsivity was significantly and positively associated with Treatment Dropout, *Wald statistic* = 4.69, *p* = 0.03. Moreover, both non-planning impulsivity, *Wald statistic* = 4.69, *p* = 0.03, and Iowa Gambling Task performance, *Wald statistic* = 4.19, *p* = 0.04, were significantly and positively associated with Relapse.

For the 12-month follow-up outcome, the model including trait and cognitive impulsivity scores showed satisfactory fit, *χ^2^* = 51.5, *d.f.* = 12, *p*<0.001, explaining 42% of variance, *Nagelkerke pseudo-R^2^* = 0.42. Inspection of parameter estimates showed that the BIS dimension of attentional impulsivity was significantly and positively associated with Treatment Dropout, *Wald statistic* = 4.21, *p* = 0.04. None of the predictors were significantly associated with Relapse (see Table 4).

**Table 4:** Multinomial regression models testing the association between trait and cognitive impulsivity and smoking cessation treatment dropout and relapse at the 3-month, 6-month and 12-month endpoints.

Impulsivity Predictors	Three months						Six months						Twelve months					
	Dropout			Relapse			Dropout			Relapse			Dropout			Relapse		
	Wald	p	95% CI	Wald	p	95% CI	Wald	p	95% CI	Wald	p	95% CI	Wald	p	95% CI	Wald	p	95% CI
Motor	1.33	.249	.95–1.24	0.000	.998	.86–1.16	1.14	.285	.93–1.30	0.01	.916	.86–1.19	4.21	.040*	1.01–1.56	1.21	.272	.92–1.36
Attention	0.54	.462	.93–1.18	2.32	.128	.97–1.24	0.77	.381	.92–1.24	3.16	.076	.99–1.29	0.05	.829	.87–1.19	0.01	.907	.88–1.16
Planning	6.02	.014*	1.03–1.25	5.34	.021*	1.02–1.25	4.69	.030*	1.01–1.26	4.19	.041*	1.01–1.23	0.86	.355	.93–1.21	2.52	.112	.98–1.22
DDT	0.63	.429	.05–3.53	2.91	.088	.75–61.72	0.000	1	.09–10.94	2.98	.084	.76–71.61	0.06	.811	.12–14.92	3.01	.083	.78–60.66
GNG	0.05	.829	.93–1.06	0.002	.964	.94–1.07	0.91	.340	.90–1.04	0.91	.341	.91–1.03	0.42	.520	.90–1.06	0.008	.931	.93–1.07
IGT	0.46	.497	.97–1.01	1.77	.184	.97–1.01	1.18	.278	.96–1.01	5.07	.024*	.95–1.0	0.49	.485	.97–1.02	2.87	.090	.96–1.00

*p<0.05. DDT, Delay Discounting Task; GNG, Go No-Go Task; IGT, Iowa Gambling Task.

### Sensitivity analyses

Bivariate logistic regression models separate for dropouts versus completers and relapse versus abstinence yielded very similar results to the main multinomial regression approach (see the Tables displaying the results of these analyses in File S1). In regards to temperament, higher novelty seeking and higher reward dependence predicted greater dropout across endpoints, and only higher persistence predicted greater relapse versus abstinence. In regards to impulsivity, higher BIS non-planning impulsivity and lower Iowa Gambling Task performance predicted greater relapse across 3 and 6 months and at 6 months respectively.

## Discussion

We demonstrate that temperament and impulsivity are significant predictors of smoking treatment outcomes. Higher scores on temperament measures of novelty seeking and reward dependence and on the impulsive trait of non-planning impulsivity were significantly associated with treatment dropout. In addition, higher scores on the temperament measure of persistence and on the impulsive trait of non-planning impulsivity, and poorer decision-making performance in the Iowa Gambling Task uniquely predicted objectively indexed smoking relapse. Sensitivity analyses further showed that higher novelty seeking and higher reward dependence are consistently associated with dropout. Moreover, higher persistence and non-planning impulsivity and poorer decision-making performance are consistently associated with relapse during later stages of treatment. These results indicate that baseline temperament and impulsivity measures are useful to prospectively predict smoking treatment outcomes, such that they can be utilised to identify clients at higher risk of poor outcomes, to match clients' profiles with adequate treatment options, or to design specific interventions for at-risk participants.

Our first finding refers to the association between novelty seeking, reward dependence and trait impulsivity with treatment drop-out. The link between novelty seeking and dropout is in fitting with previous evidence demonstrating that high sensation seeking is associated with poorer treatment response to smoking cessation motivational interventions [40]. This finding is also reminiscent of the broader substance use treatment literature, whereby several studies have shown that alcohol and substance using clients scoring higher on novelty seeking are more susceptible to dropout as soon as the novelty of treatment fades out [7], [15]. We also showed, for first time, that the temperament dimension of reward dependence is linked to smoking treatment dropout. Interestingly, reward dependence was the only temperament measure correlated with steeper discounting of delayed reward, which has been linked to smoking treatment outcomes in previous studies [13], [41] and which showed a sizeable (although non-significant) contribution to dropout in this sample. Moreover, we showed that non-planning and attentional dimensions of impulsivity are linked to higher treatment dropout. This finding was expected, considering that the beneficial outcomes of smoking interventions are not immediate [42] such that adequate focus on long-term goals is required to continue with treatment. Our results regarding prediction of dropout suggest different routes for improving engagement in smoking cessation. Since temperament is regarded as a stable disposition [25], temperament-based treatment matching could be utilised to allocate clients to tailored treatment options. For example, novelty seekers are likely to stick to programs with varied stimulation and challenges. Similarly, reward dependent clients could get unique benefit from contingency management interventions [43]. Although non-planning impulsivity is also regarded as a trait, novel evidence has revealed that trait dispositions are malleable to self-regulation interventions [44].

Our second finding refers to the association between persistence, non-planning impulsivity and decision-making with smoking relapse. Persistence was originally defined as perseverance despite frustration [4] which might be viewed as an advantageous disposition for smoking cessation, and specifically for early stages of treatment. However, high persistence scores are associated with resistance to extinction of previously rewarded behaviours [45]. Therefore, highly persistent clients are purportedly more prone to perseverate on stimulus-bound instrumental behaviours, and therefore more vulnerable to relapse in the long-term. Non-planning impulsivity refers to lack of forethought about the long-term outcomes of acts and decisions [11]. Previous studies had shown that overall trait impulsivity levels are associated with smoking relapse following treatment [13], [46] but this is the first study to show that this particular dimension is significantly associated with relapse. Moreover, disadvantageous (reward-driven, risk-insensitive) decision-making in the Iowa Gambling Task was also significantly predictive of smoking relapse, in agreement with findings from animal studies [47] and human studies predicting relapse in other substance using populations [19], [20]. Interestingly, the three significant predictors of smoking relapse (persistence, non-planning impulsivity and decision-making) share an overlapping neural substrate in the medial orbitofrontal cortex [48]–[51]. This region is specialised in integrating emotional states with stimulus-outcome representations, and is therefore critical to estimate the risk and to anticipate the consequences of our decisions. Therefore, our results suggest that tailored interventions directed to target these mechanisms, such as self-regulation training or episodic future thinking [52], [53], could be effective to improve treatment outcomes in smoking cessation.

This study shows that novelty seeking, reward dependence and non-planning impulsivity are significant predictors of smoking treatment dropout, whereas persistence, non-planning impulsivity and decision-making are significant predictors of smoking relapse. Harm avoidance and motor impulsivity do not significantly predict poor smoking treatment outcomes, and delay discounting showed only a trend in predicting retention. The strength of prediction effects is higher for impulsivity than for temperament measures. Since impulsivity is as well more malleable than temperamental dispositions, our findings suggest that tailored interventions aimed to enhance impulse control and to direct motivation towards long-term goals may increase efficacy of smoking treatment programs. The main strengths of this study include the relatively large sample size, the multidimensional assessment of trait and cognitive domains, and the objective measurement of relapse. Our results should be as well understood in the context of relevant limitations. First, the study design was naturalistically embedded in the context of a health promotion intervention (i.e., individuals who dropped-out at an earlier stage could re-engage at a later stage). Therefore, the different endpoints cannot be interpreted from a longitudinal perspective, but as discrete time-points. Future studies are warranted to examine whether the observed associations stand in a purely longitudinal design using survival analyses approaches. Further, treatment participants were all employees of the same institution, the University of Granada, and there may be a concern that the sample is not representative of clinical populations. However, since smoking is a broad community-spread problem, and participants belonged to different strata of the University make-up (academics, professionals, and administrative staff), the sample is sufficiently diverse to be representative of the general population. Another potential limitation is the exclusion of participants with Axis I disorders, which probably precludes inclusion of individuals in the upper extreme of the constructs examined (e.g., novelty seeking, impulsivity). However, this only means that the sample is more representative of smoking cessation outcomes in the community versus specific clinical settings. Moreover, even though abstinence was cross-validated with self-report and haemoglobin levels, both assessment methods are subjected to biases, such as reliability of retrospective reports (self-report) and time-limited scope (co-oximetry haemoglobin levels). Multiple tests may raise concerns about Type I error, but results were highly consistent with hypotheses, and the main findings were supported by the sensitivity analyses. Special caution must be taken with unpredicted results, such as the association between attentional impulsivity and long-term relapse. Collectively, our findings demonstrate that temperament is essential for prediction of smoking treatment retention, and that both temperament and impulsivity dimensions associated with long-term based decision-making (persistence, non-planning impulsivity, cognitive-affective decision making) are important for prediction of smoking relapse.

## Supporting Information

File S1 **Supporting tables. Tables S1–S12.**
(DOCX)
