# Supplementary material for: Temperament and Impulsivity Predictors of Smoking Cessation Outcomes
Source: PLoS One. 2014 Dec 4;9(12):e112440. doi: 10.1371/journal.pone.0112440 (PMC4256301; doi:10.1371/journal.pone.0112440)
Supplement: File S1 — Supporting tables. Tables S1–S12. (DOCX) [file pone.0112440.s001.docx]

**SUPPLEMENTARY MATERIALS FOR**

**Temperament and impulsivity predictors of smoking cessation outcomes**

Francisca López -Torrecillas^1,2^**,** José Cesar Perales^2,3,4^, Ana Nieto-Ruiz^5^**,** AntonioVerdejo-García^1,4,6^

^1^Department of Personality, Assessment and Psychological Treatment, University of Granada, Spain.

^2^Center Research Mind Brain and Behavior (CIMCYC), University of Granada, Spain;

^3^Department of Experimental Psychology, University of Granada, Spain.

^4^Red de Trastornos Adictivos, University of Granada, Spain.

^5^Occupational Medicine Area (Prevention Service), University of Granada, Spain.

^6^School of Psychological Sciences, Monash University, Australia.

Corresponding author:

Francisca López Torrecillas, Ph. D.

Departamento de Personalidad, Evaluación y Tratamiento Psicológico

Universidad de Granada

Campus de Cartuja

18071, Granada, Spain

[fcalopez@ugr.es](mailto:fcalopez@ugr.es)

+34 654053842

**Supplementary Tables S1 to S3: Binary logistic regression models for temperament and completion versus dropout**

Table S1: Binomial regression models testing the association between TCI temperament dimensions and smoking cessation treatment completion versus dropout at the 3-month endpoint (77.9 % correctly predicting).

| Temperament Predictors | **B** | **E.T.** | **Wald** | ***p*** | **OR** | **95% IC** | |
| --- | --- | --- | --- | --- | --- | --- | --- |
| Novelty seeking | .057 | .019 | 9.010 | .003* | 1.059 | 1.020 | 1.099 |
| Harm avoidance | .003 | .012 | .073 | .788 | 1.003 | .980 | 1.027 |
| Reward dependence | .035 | .016 | 4.910 | .027* | 1.036 | 1.004 | 1.068 |
| Persistence | .017 | .014 | 1.473 | .225 | 1.017 | .990 | 1.044 |
| Constant | -13.254 | 3.125 | 17.987 | .000 | .000 |  |  |

*p<0.05

Table S2: Binomial regression models testing the association between TCI temperament dimensions and smoking cessation treatment completion versus dropout at the 6-month endpoint (72.2 % correctly predicting).

| Temperament Predictors | **B** | **E.T.** | **Wald** | ***p*** | **OR** | **95% IC** | |
| --- | --- | --- | --- | --- | --- | --- | --- |
| Novelty seeking | .056 | .020 | 8.073 | .004* | 1.058 | 1.018 | 1.100 |
| Harm avoidance | .002 | .013 | .022 | .883 | 1.002 | .977 | 1.028 |
| Reward dependence | .035 | .018 | 3.997 | .046* | 1.036 | 1.001 | 1.072 |
| Persistence | .026 | .015 | 2.970 | .085 | 1.026 | .996 | 1.056 |
| Constant | -14.000 | 3.345 | 17.514 | .000 | .000 |  |  |

*p<0.05

Table S3: Binomial regression models testing the association between TCI temperament dimensions and smoking cessation treatment completion versus dropout at the 12-month endpoint (77.7 % correctly predicting).

| Temperament Predictors | **B** | **E.T.** | **Wald** | ***p*** | **OR** | **95% IC** | |
| --- | --- | --- | --- | --- | --- | --- | --- |
| Novelty seeking | .057 | .020 | 7.667 | .006* | 1.058 | 1.017 | 1.102 |
| Harm avoidance | .007 | .013 | .304 | .581 | 1.007 | .981 | 1.034 |
| Reward dependence | .037 | .018 | 4.095 | .043* | 1.038 | 1.001 | 1.075 |
| Persistence | .019 | .016 | 1.491 | .222 | 1.020 | .988 | 1.052 |
| Constant | -14.062 | 3.400 | 17.106 | .000 | .000 |  |  |

*p<0.05

**Supplementary Tables S4 to S6: Binary logistic regression models for temperament and relapse versus abstinence**

Table S4: Binomial regression models testing the association between TCI temperament dimensions and smoking cessation treatment abstinence versus relapse at the 3-month endpoint (75.9 % correctly predicting).

| Temperament Predictors | **B** | **E.T.** | **Wald** | ***p*** | **OR** | **95% IC** | |
| --- | --- | --- | --- | --- | --- | --- | --- |
| Novelty seeking | .013 | .018 | .508 | .476 | 1.013 | .978 | 1.049 |
| Harm avoidance | .013 | .014 | .978 | .323 | 1.014 | .987 | 1.041 |
| Reward dependence | .008 | .016 | .299 | .585 | 1.009 | .978 | 1.040 |
| Persistence | .018 | .014 | 1.546 | .214 | 1.018 | .990 | 1.047 |
| Constant | -6.711 | 3.335 | 4.050 | .044 | .001 |  |  |

*p<0.05

Table S5: Binomial regression models testing the association between TCI temperament dimensions and smoking cessation treatment abstinence versus relapse at the 6-month endpoint (61.3 % correctly predicting).

| Temperament Predictors | **B** | **E.T.** | **Wald** | ***p*** | **OR** | **95% IC** | |
| --- | --- | --- | --- | --- | --- | --- | --- |
| Novelty seeking | .017 | .018 | .900 | .343 | 1.017 | .982 | 1.054 |
| Harm avoidance | .019 | .014 | 1.638 | .201 | 1.019 | .990 | 1.048 |
| Reward dependence | .003 | .016 | .040 | .841 | 1.003 | .973 | 1.034 |
| Persistence | .037 | .016 | 5.786 | .016* | 1.038 | 1.007 | 1.070 |
| Constant | -8.548 | 3.589 | 5.672 | .017 | .000 |  |  |

*p<0.05

Table S6: Binomial regression models testing the association between TCI temperament dimensions and smoking cessation treatment abstinence versus relapse at the 12-month endpoint (60.7 % correctly predicting).

| Temperament Predictors | **B** | **E.T.** | **Wald** | ***p*** | **OR** | **95% IC** | |
| --- | --- | --- | --- | --- | --- | --- | --- |
| Novelty seeking | .003 | .019 | .024 | .876 | 1.003 | .967 | 1.040 |
| Harm avoidance | .014 | .014 | 1.008 | .315 | 1.014 | .987 | 1.042 |
| Reward dependence | .003 | .016 | .028 | .867 | 1.003 | .973 | 1.034 |
| Persistence | .019 | .015 | 1.682 | .195 | 1.019 | .990 | 1.049 |
| Constant | -4.219 | 3.212 | 1.725 | .189 | .015 |  |  |

*p<0.05.

**Supplementary Tables S7 to S9: Binary logistic regression models for impulsivity and completion versus dropout**

Table S7. Binomial regression models testing the association between trait and cognitive impulsivity dimensions and smoking cessation treatment completion versus dropout at the 3-month endpoint (85 % correctly predicting).

| Impulsivity Predictors | **B** | **E.T.** | **Wald** | ***p*** | **OR** | **95% IC** | |
| --- | --- | --- | --- | --- | --- | --- | --- |
| Motor | .071 | .052 | 1.877 | .171 | 1.074 | .970 | 1.189 |
| Attention | .022 | .054 | .165 | .685 | 1.022 | .919 | 1.136 |
| Non-Planning | .087 | .046 | 3.660 | .056 | 1.091 | .998 | 1.193 |
| DDT | -1.558 | .964 | 2.610 | .106 | .211 | .032 | 1.394 |
| GNG | -.007 | .030 | .052 | .819 | .993 | .937 | 1.053 |
| IGT | -.002 | .010 | .066 | .798 | .998 | .979 | 1.016 |
| Constant | -3.578 | .979 | 13.353 | .000 | .028 |  |  |

*p<0.05. DDT, Delay Discounting Task; GNG, Go No-Go Task; IGT, Iowa Gambling Task

Table S8. Binomial regression models testing the association between trait and cognitive impulsivity dimensions and smoking cessation treatment completion versus dropout at the 6-month endpoint (80.5 % correctly predicting).

| Impulsivity Predictors | **B** | **E.T.** | **Wald** | ***p*** | **OR** | **95% IC** | |
| --- | --- | --- | --- | --- | --- | --- | --- |
| Motor | .076 | .057 | 1.771 | .183 | 1.079 | .965 | 1.206 |
| Attention | .019 | .058 | .112 | .738 | 1.020 | .910 | 1.142 |
| Non-Planning | .072 | .048 | 2.280 | .131 | 1.075 | .979 | 1.180 |
| DDT | -1.045 | .979 | 1.140 | .286 | .352 | .052 | 2.395 |
| GNG | -.016 | .031 | .273 | .601 | .984 | .925 | 1.046 |
| IGT | .000 | .010 | .003 | .960 | 1.000 | .982 | 1.019 |
| Constant | -3.436 | .973 | 12.461 | .000 | .032 |  |  |

*p<0.05. DDT, Delay Discounting Task; GNG, Go No-Go Task; IGT, Iowa Gambling Task

Table S9. Binomial regression models testing the association between trait and cognitive impulsivity dimensions and smoking cessation treatment completion versus dropout at the 12-month endpoint (77.7 % correctly predicting).

| Impulsivity Predictors | **B** | **E.T.** | **Wald** | ***p*** | **OR** | **95% IC** | |
| --- | --- | --- | --- | --- | --- | --- | --- |
| Motor | .132 | .083 | 2.520 | .112 | 1.141 | .970 | 1.342 |
| Attention | .038 | .063 | .364 | .547 | 1.039 | .918 | 1.176 |
| Non-Planning | .013 | .059 | .047 | .829 | 1.013 | .903 | 1.136 |
| DDT | -.925 | .986 | .879 | .348 | .397 | .057 | 2.739 |
| GNG | -.025 | .034 | .547 | .460 | .975 | .912 | 1.043 |
| IGT | .002 | .010 | .052 | .819 | 1.002 | .983 | 1.022 |
| Constant | -3.632 | 1.049 | 11.984 | .001 | .026 |  |  |

*p<0.05. DDT, Delay Discounting Task; GNG, Go No-Go Task; IGT, Iowa Gambling Task

**Supplementary Tables S10 to S12: Binary logistic regression models for impulsivity and relapse versus abstinence**

Table 10. Binomial regression models testing the association between trait and cognitive impulsivity dimensions and smoking cessation treatment abstinence versus relapse at the 3-month endpoint (80.6 % correctly predicting).

| Impulsivity Predictors | **B** | **E.T.** | **Wald** | **p** | **OR** | **95% IC** | |
| --- | --- | --- | --- | --- | --- | --- | --- |
| Motor | -.020 | .087 | .051 | .821 | .981 | .827 | 1.162 |
| Attention | .121 | .072 | 2.809 | .094 | 1.128 | .980 | 1.299 |
| Non-Planning | .117 | .055 | 4.440 | .035* | 1.124 | 1.008 | 1.253 |
| DDT | 1.429 | 1.218 | 1.376 | .241 | 4.175 | .383 | 45.473 |
| GNG | -.004 | .031 | .021 | .886 | .996 | .936 | 1.059 |
| IGT | -.012 | .011 | 1.177 | .278 | .988 | .967 | 1.010 |
| Constant | -5.290 | 1.220 | 18.792 | .000 | .005 |  |  |

*p<0.05. DDT, Delay Discounting Task; GNG, Go No-Go Task; IGT, Iowa Gambling Task

Table 11. Binomial regression models testing the association between trait and cognitive impulsivity dimensions and smoking cessation treatment abstinence versus relapse at the 6-month endpoint (76.3 % correctly predicting).

| Impulsivity Predictors | **B** | **E.T.** | **Wald** | ***p*** | **OR** | **95% IC** | |
| --- | --- | --- | --- | --- | --- | --- | --- |
| Motor | -.023 | .090 | .066 | .798 | .977 | .819 | 1.165 |
| Attention | .151 | .077 | 3.847 | .050* | 1.163 | 1.000 | 1.351 |
| Non-Planning | .114 | .055 | 4.238 | .040* | 1.121 | 1.005 | 1.249 |
| DDT | 1.331 | 1.261 | 1.115 | .291 | 3.786 | .320 | 44.810 |
| GNG | -.031 | .031 | 1.003 | .317 | .969 | .911 | 1.031 |
| IGT | -.024 | .012 | 4.208 | .040* | .976 | .954 | .999 |
| Constant | -4.568 | 1.164 | 15.394 | .000 | .010 |  |  |

*p<0.05. DDT, Delay Discounting Task; GNG, Go No-Go Task; IGT, Iowa Gambling Task

Table 12. Binomial regression models testing the association between trait and cognitive impulsivity dimensions and smoking cessation treatment abstinence versus relapse at the 12-month endpoint (72.6 % correctly predicting).

| Impulsivity Predictors | **B** | **E.T.** | **Wald** | ***p*** | **OR** | **95% IC** | |
| --- | --- | --- | --- | --- | --- | --- | --- |
| Motor | .066 | .097 | .473 | .492 | 1.069 | .884 | 1.291 |
| Attention | .036 | .074 | .231 | .631 | 1.036 | .896 | 1.198 |
| Non-Planning | .096 | .057 | 2.863 | .091 | 1.101 | .985 | 1.232 |
| DDT | 1.252 | 1.174 | 1.138 | .286 | 3.499 | .350 | 34.926 |
| GNG | -.012 | .034 | .131 | .717 | .988 | .925 | 1.055 |
| IGT | -.016 | .011 | 2.339 | .126 | .984 | .964 | 1.005 |
| Constant | -3.450 | 1.046 | 10.875 | .001 | .032 |  |  |

*p<0.05. DDT, Delay Discounting Task; GNG, Go No-Go Task; IGT, Iowa Gambling Task
